# Effects of soil properties and carbon substrates on bacterial diversity of two sunflower farms

**DOI:** 10.1186/s13568-022-01388-9

**Published:** 2022-04-23

**Authors:** Blessing Chidinma Nwachukwu, Ayansina Segun Ayangbenro, Olubukola Oluranti Babalola

**Affiliations:** Food Security and Safety Focus Area, Faculty of Natural and Agricultural Science, North-West University, Private Mail Bag X2046, Mmabatho, 2735 South Africa

**Keywords:** Bacterial community, Functional profile, Sustainable agriculture, 16S amplicon sequencing

## Abstract

The sustainable production of sunflower (*Helianthus annuus*) is crucial and one way to accomplish this feat is to have an understanding of the beneficial bacteria of sunflower rhizosphere. Similarly, the respiratory response of these bacteria needs to be studied to understand their roles in the ecosystem. This study was therefore conceptualized to gain insights into the effects of soil properties and carbon substrate utilization on bacterial community diversity of sunflower rhizosphere grown in Ditsobottla and Kraaipan, North West Province, South Africa. Extracted DNA from sunflower rhizosphere and bulk soils was subjected to 16S amplicon sequencing. Significant differences were observed in the alpha and beta diversities of the soil bacterial communities (p < 0.05). At the order level, among all the bacterial taxa captured in the farms, *Bacillales* were the most dominant. The abundance of *Lactobacillales*, *Bacillales*, *Rhizobiales*, *Enterobacteriales*, *Burkholderiales*, *Flavobacteriales*, *Sphingomonadales*, *Myxococcales*, and *Nitrosomonadales* obtained from Ditsobottla rhizosphere soil (R1) was positively influenced by organic matter (OM), while the abundance of *Planctomycetales*, *Cytophagales*, *Gemmatimonadales*, *Nitrospirales* and *Caulobacteriales* from Kraaipan rhizosphere soil (R2) was positively influenced by total N and pH. Bacterial communities of all the soil samples utilized the different carbon substrates (three amino acids, six carbohydrates, and three carboxylic acids) as an energy source. Significant differences (p < 0.05) were only observed in tryptophan and methionine amended soils. Unclassified bacteria were also captured in this study, such bacteria can further be harnessed for sustainable production of sunflower and other agricultural crops.

**Supplementary information:**

The online version contains supplementary material available at 10.1186/s13568-022-01388-9.

## Introduction

Over a decade, studies have revealed that plant-microbial interactions are crucial for plant growth and health, and these interactions are of immense importance in comprehending nature conservation and agricultural sustainability (Nwachukwu and Babalola 2021). Similarly, recent attempts to understand the belowground activities have shown that the rhizosphere bacterial communities play a key role in improving ecosystem functions and plant yield (Agomoh et al. 2020; Igiehon and Babalola 2018). The richness and diversity of rhizosphere bacterial communities significantly contribute to the resilience and resistance of the ecosystem to biotic and abiotic stresses (Shrestha et al. 2019). Interestingly, awareness into plant–soil interactions and processes are complex and still require further studies (Yee et al. 2021).

Soil microorganisms play important roles in many soil processes, including nutrient acquisition, nitrogen and carbon acquisition, and transformation of plant deposits to soil organic matter (Jiang et al. 2021; Enebe and Babalola 2021). In plants, nutrient distributions and accessibility differ significantly depending on the soil type, plant species, and plant age (Nwachukwu and Babalola 2021; Lambers et al. 2008). Generally, plants influence the rhizosphere bacterial communities through the release of metabolites that stimulate or reduce the proliferation of a particular species of the microbiota, thus creating an environment favorable for the plant species (Nwachukwu et al. 2021; Igiehon et al. 2019). Other plant species harbors species-specific microorganisms that are strictly linked with them in the rhizosphere (Yee et al. 2021). Rhizosphere bacterial communities likewise show an advanced level of host interaction and specificity with plant species (Schlatter et al. 2019).

On the contrary, cropping system and agricultural practices have a strong influence on soil microbial diversity, crop benefits and soil properties (Zhang et al. 2019). A lot of studies have assessed soil bacterial diversity, limited studies have, however, investigated the impact of carbon substrate utilization on their functions. Nevertheless, considering the multifaceted dynamics that regulate rhizosphere bacterial communities, the fundamental significance and functions of rhizosphere microbiome are still not fully understood (Shrestha et al. 2019).

Furthermore, physicochemical properties of soils and other abiotic components can regulate the interactions between soil bacterial communities and plants (Igiehon et al. 2019). The ability of soil bacterial communities to breakdown numerous organic compounds helps to control the transformation rate of these compounds. Therefore, for proper functioning of soils, the structural and functional diversity of bacterial communities are of enormous significance (Chukwuneme et al. 2021; Babalola et al. 2021). Insight into carbon substrate utilization of the bacterial communities inhabiting sunflower rhizosphere and bulk soils is also important to comprehend how these ecosystems function. To the best of our knowledge, limited studies exist on the processes and effects of different carbon respiration on the diversity, structural and functional characteristics of the rhizosphere microbiome associated with sunflower plants. The respiratory response of soil microorganisms to environmentally relevant substrates, especially those involved in plant-bacterial activities need to be understood to reveal the role played by diverse soil microorganisms and their functional diversities. This study, therefore, aimed to unveil the influence of rhizosphere carbon respiration on the structural and functional attributes used by the rhizosphere bacterial community associated with sunflower plants using amplicon sequencing, and community level physiological profile (CLPP). We hypothesized that the bacterial community structure and CLPP of sunflower rhizosphere soils would be different from bacterial community structure and CLPP of their corresponding bulk soils. These projected variations in bacterial community structure and carbon utilization between the soil habitats may be alluded to a significant increase in the amount of root exudates, plant-bacterial interactions, and greater utilization of highly structurally complex carbon substrates in the rhizosphere soil compared to their corresponding bulk soils.

## Materials and methods

### Farm history and sample collection

This study was performed in the North West Province of South Africa. The province has a summer period between December and February characterized with short thundershowers in the afternoon. The mean rainfall is between 300 and 700 mm per annum, while during summer and winter the temperatures are between 22 and 34 °C. The annual mean temperature during winter (June–August) is between 2 and 20 °C, while autumn (March–May) and spring (September–November) annual temperatures are mainly between 13 and 25 °C and 19–30 °C respectively.

The two major sunflower plant farms in Ditsobottla also called Sheila (26^o^ 2′41.202″ S 25^o^ 57′ 47. 49″ E) and Kraaipan (26^o^ 17′24.186″ S 25^o^ 13′33.258″ E) were cautiously selected based on the agricultural practices of the farms (Additional file 2: Table S1). Approval was obtained from the farm owners. Sunflower seed (Pen 7011 Pannar) was the cultivar planted on both farms. Over the years, both farms have history of fertilizer (NPK 15:8:4) application.

Precisely, after 8 weeks of cultivation, rhizosphere and bulk (control) soils were collected from both farms. Two replicate soil samples each were obtained for Ditsobottla and Kraaipan rhizosphere soils (R1 and R2 respectively), and each replicate was composed of soils collected from 20 plants after germination at a depth of 0–15 cm on different plots having radius of 3 cm away from the plants, and afterward pooled together to make a composite sample as described by Oberholster et al. (2018). Similarly, Ditsobottla and Kraaipan bulk soils (B1 and B2 respectively) were collected from the surrounding soil at a distance of 10 m away from the sunflower rhizospheres.

The soil samples were collected into sterile plastic bags, then transferred into an ice packed container and conveyed to the laboratory immediately. A mesh sieve (2-mm pore size) was used to sieve the soil samples, while the sieved soil samples were divided into three portions: one portion was used for 16S amplicon sequencing, which was stored at – 20 °C, the second portion was stored at 4 °C for physiochemical analyses, while the third portion was used for CLPP.

### Soil physiochemical analyses

A Jenway 3520 pH-meter (Cole-Parmer Instruments, Staffordshire, UK) was used to determine soil pH after mixing 2 g of the soil in 10 ml deionized water. Soil moisture content was quantified by oven-drying the soil samples at 105 °C for 24 h (Colombo et al. 2016). The total nitrogen was determined using dry combustion method, while Walkley Black method was used to ascertain the organic carbon (Walkley and Black 1934; Jacoby et al. 2017).

### Metagenome DNA extraction and amplicon sequencing

Zymo DNA isolation kit (Zymo Research, Irvine, USA) was used for the extraction of the DNA following manufacturer’s instruction. The extracted DNA samples were sent to MR DNA Laboratory (Texas, USA) for 16S amplicon sequencing. The 16S rRNA gene variable region V4 for bacterial community was sequenced using an Illumina MiSeq sequencer (Illumina NovaSeq 6000 system). The PCR primers 515 F and 806R were used, and then paired-ends reads of 312 bp were obtained.

### Annotation, data and statistical analyses

The forward and reverse sequences were obtained after sequencing was performed on the Illumina MiSeq sequencer using a paired-end technique. The raw sequence reads were uploaded into MG-RAST, an online server (Meyer et al. 2008). After joining the paired ends on the MG-RAST to generate consensus sequences, quality control (QC) processes were carried out, and the sequence reads were pre-processed (to remove the artificial sequences, ambiguous base pairs and host specific-species sequences), followed by filtering of the read length. Then, the sequences were denoised and screened for the presence of chimeras. The processed sequences were annotated using BLASTn (Mohammed et al. 2020) against RDPII and NCBI databases (Garcia-Mazcorro et al. 2019), permitting non-redundant integration of numerous databases. The RDP was used to classify the bacteria.

Further analyses were not conducted on sequences that failed annotation. The normalized data option of MG-RAST was used to reduce the effect of experimental error/noise. The bacterial abundance values which were assembled according to the taxa, and unclassified bacteria were reserved for statistical purposes. Afterward, the taxa abundance values were transformed into percentages. Binning was carried out on the analogous sequences into operational taxonomic units (OTUs). OTUs were clustered at 3% divergence (97% similarity). Equitability_J, Fisher_alpha, Berger-Parker and Chao-1 were used to estimate species richness. Taxonomic richness was expressed as OTU number. Furthermore, statistical analysis was carried out using the mean of the relative abundance for all the replicates of each sampling site. The sequences have been deposited on the National Center for Biotechnology Information SRA dataset under the accession number PRJNA672856.

### Determination of carbon substrate utilization by soil bacterial components using CLPP technique —MicroPlate™

The bacterial community level physiological profiles of the soil samples were determined using the MicroResp™ method (Moscatelli et al. 2018). Soil samples were adjusted to 40% of their maximum water holding capacity and loaded into 96-well of 1.2 ml deep-well microplate. Prior to carrying out MicroResp™ method, 0.4 g of soil was distributed to each well and incubated in the dark at 25 °C for 5 days.

Eleven (11) different carbon substrates, consisting of 3 amino acids (L-methionine, L-tyrosine, S-tryptophan), 6 carbohydrates (D-galactose, D-glucose, d-fructose, d-maltose, Sucrose) and 3 carboxylic acids (d-pantothenic, citric acid, malic acid) were used to determine the physiological profiles, while distilled water served as the control. Sterile deionized water was used to measure individual basal respiration. Each substrate dissolved in sterile deionized water was added to 4 replicated wells. Detection plates containing cresol red (12.5 µg/ml), potassium chloride (150 mM) and sodium bicarbonate (2.5 mM) were read at absorbance wavelength 570 nm, and immediately placed on the MicroResp™ seal, and then incubated at 25 °C in the dark for 6 h as recommended in the instruction manual. After incubation, the modification in optical density (OD) was determined on a spectrophotometer microplate reader (AccuReader M965+, Taipei, Taiwan) at a wavelength of 570 nm. The rate of individual CO_2_ respiration per gram of soil was calculated using the formula provided in the MicroResp™ manual (James Hutton Ltd, UK). Total respiration rate was obtained by following the method of Colombo et al. (2016) with little modification.

### Statistical analyses

Microsoft Excel Software was used to analyze the abundance and distribution of the key rhizosphere bacterial communities at the class and order level. Also, Shinyheatmap online tool (www1.heatmapper.ca/expression/) was used for the plotting of heatmaps using the relative abundance values. Equitability_J, Fisher_alpha, Berger-Parker and Chao-1 indices for diversity assessment were used for samples across rhizosphere and bulk soil samples, and the comparison of these indices was performed using Kruskal–Wallis test. The analyses were carried out using PAST version 3.20 (Hammer et al. 2001). Beta (β) diversity of the bacterial communities of the samples was determined using the Euclidean-based principal coordinate analysis (PCoA) and ANOSIM through 999 permutations. *p* < 0.05 was considered statistically significant for all datasets. Both PCoA and Principal Component Analysis (PCA) graphs were used to evaluate the relationship between bacterial communities and the measured physiochemical parameters, which were plotted using CANOCO version 5.0 (Microcomputer Power, Ithaca, NY). The effects of pH, total N and OM on the bacterial community distribution were determined using Canonical Correspondence analysis (CCA). Graphs and one-way ANOVA for functional measurements were achieved using GraphPad Prism 7 (GraphPad Software, California).

## Results

### Amplicon metagenome sequencing, quality control and protein annotation

An estimated mean number of sequences uploaded were 73,877,515 (R1) and 80,404,565 (R2) sequence reads for sunflower rhizosphere soils and 50,642,798 (B1) and 74,956,208 (B2) sequence reads for bulk soil samples. After performing quality control (QC) using MG-RAST software, the estimated sequence were 8,362,450 (R1) with a mean G + C content of 56%, and R2 had a mean sequence of 8,310,524 with a mean G + C content of 55.5%, while the mean sequence of B1 sample was 5,824,673 with a mean G + C content of 54.5%. Whereas, B2 had a mean sequence of 9,525,300 with a mean G + C content of 57%. Additionally, 29,112 (R1) and 272,663 (R2) sequence reads contained predicted proteins with unknown functions from the sunflower rhizosphere soils, while 173,859 (B1) and 254,030 (B2) sequence reads contained predicted proteins with unidentified functional categories from sunflower bulk soil samples.

**Fig. 1:**
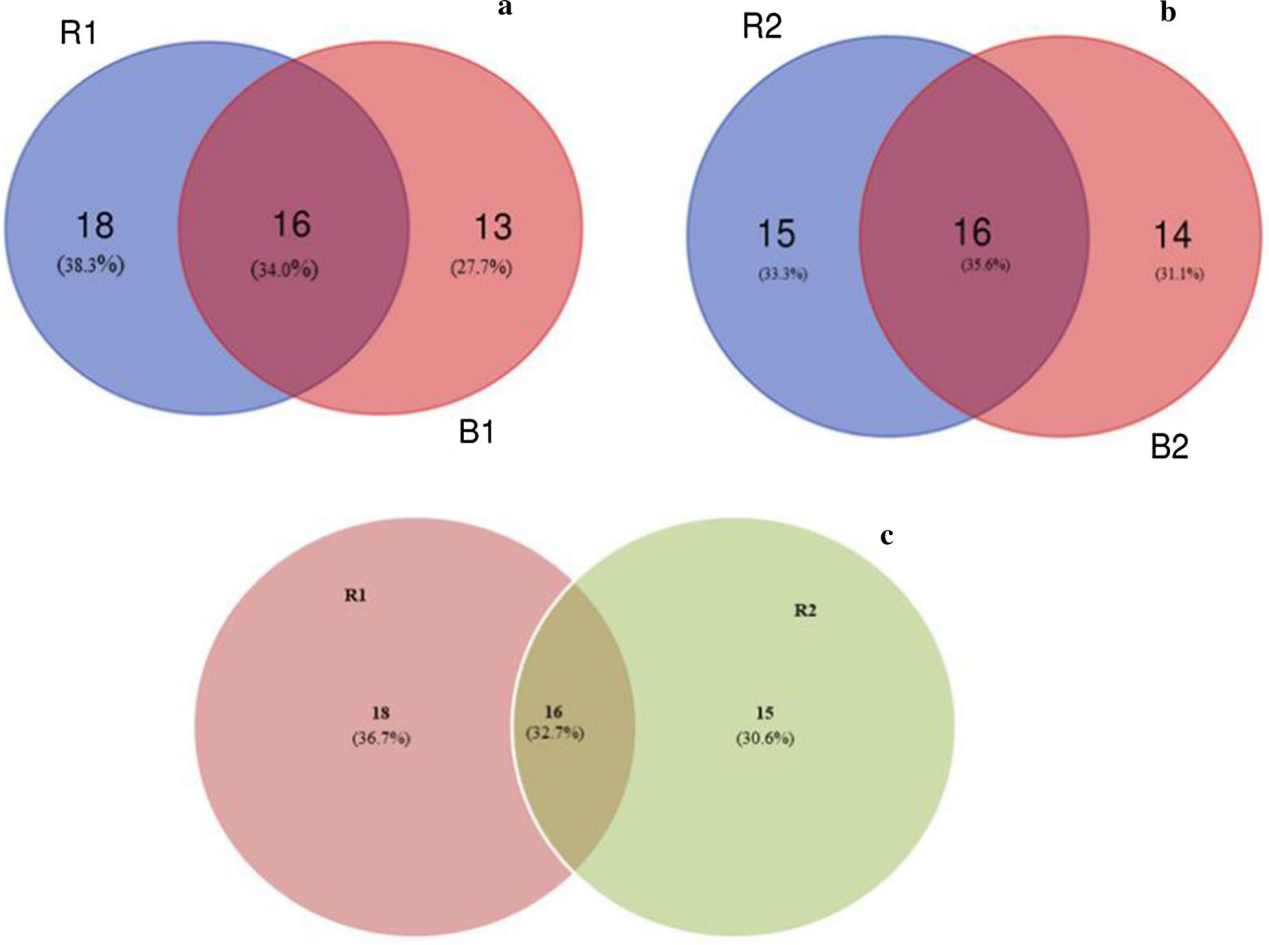
Venn diagram of shared operation taxonomic units between the bacterial components (at the order level) of sunflower rhizosphere and bulk soils obtained from Ditsobottla and Kraaipan farms. *R1* Ditsobottla rhizosphere soil; *B1* Ditsobottla bulk soil; *R2* Kraaipan rhizosphere soil; *B2*- Kraaipan bulk soil

OTU sequences were clustered at a similarity of 97% and the abundant values for R1, R2, B1 and B2 were obtained. A Venn diagram http://bioinformatics.psb.ugent.be/cgi-bin/liste/Venn/calculate_venn.htpl (Oliveros 2017) (Fig. 1) revealed that R1 and B1 shared 34.0% of OTUs, while R2 and B2 shared 35.6% of OTUs. However, 32.7% of OTUs was shared between R1 and R2.

### Alpha and beta diversity assessment of the bacterial communities across the sampling sites

**Fig. 2:**
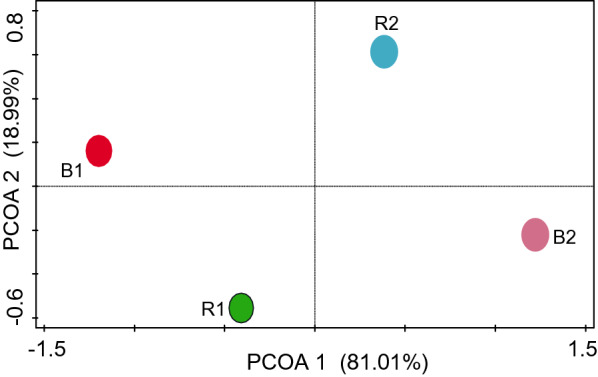
PCoA of the bacterial components (at the order level) of sunflower rhizosphere and bulk soils obtained from Ditsobottla and Kraaipan farms using Canonical correspondence analysis. *PCoA* principal coordinates analysis; *R1* Ditsobottla rhizosphere soil; *B1* Ditsobottla bulk soil; *R2* Kraaipan rhizosphere soil; *B2* Kraaipan bulk soil

The diversity of the structural categories was examined using the equitability_J, Fisher_alpha, Berger-Parker and Chao-1, and the results revealed that they differ significantly (*p <* 0.05) (Table 1). Through Kruskal-Wallis evaluation, the level of the differences in structural diversity across the sites was estimated and was found to be significant (*p* = 0.01). The PCoA graph revealed a clear difference in the relative abundance of bacterial groups between R1 and B1 sites as compared to R2 and B2 sites (Fig. 2). One-way ANOSIM was used to test for similarity across the sites. The results showed a significant difference in the identified bacterial communities from all samples across the cropping sites (R = 1.167 and *p* = 0.01).

**Table 1:** Alpha diversity indices of the bacterial components of sunflower rhizosphere and bulk soils

Alpha diversity indices	Soil type				*p* value
	R1	B1	R2	B2	
Equitability_J	0.4894	0.4366	0.5861	0.5579	0.01
Fisher_alpha	11.9	11.51	10.67	9.571	
Berger-Parker	0.512	0.4373	0.3552	0.4314	
Chao-1	23	22	22	22	

*R1* Ditsobottla rhizosphere soil; *B1* Ditsobottla bulk soil; *R2* Kraaipan rhizosphere soil; *B2* Kraaipan bulk soil; *p* probability value

### Bacterial structural composition at the class and order level across the rhizosphere and bulk soil samples

**Fig. 3:**
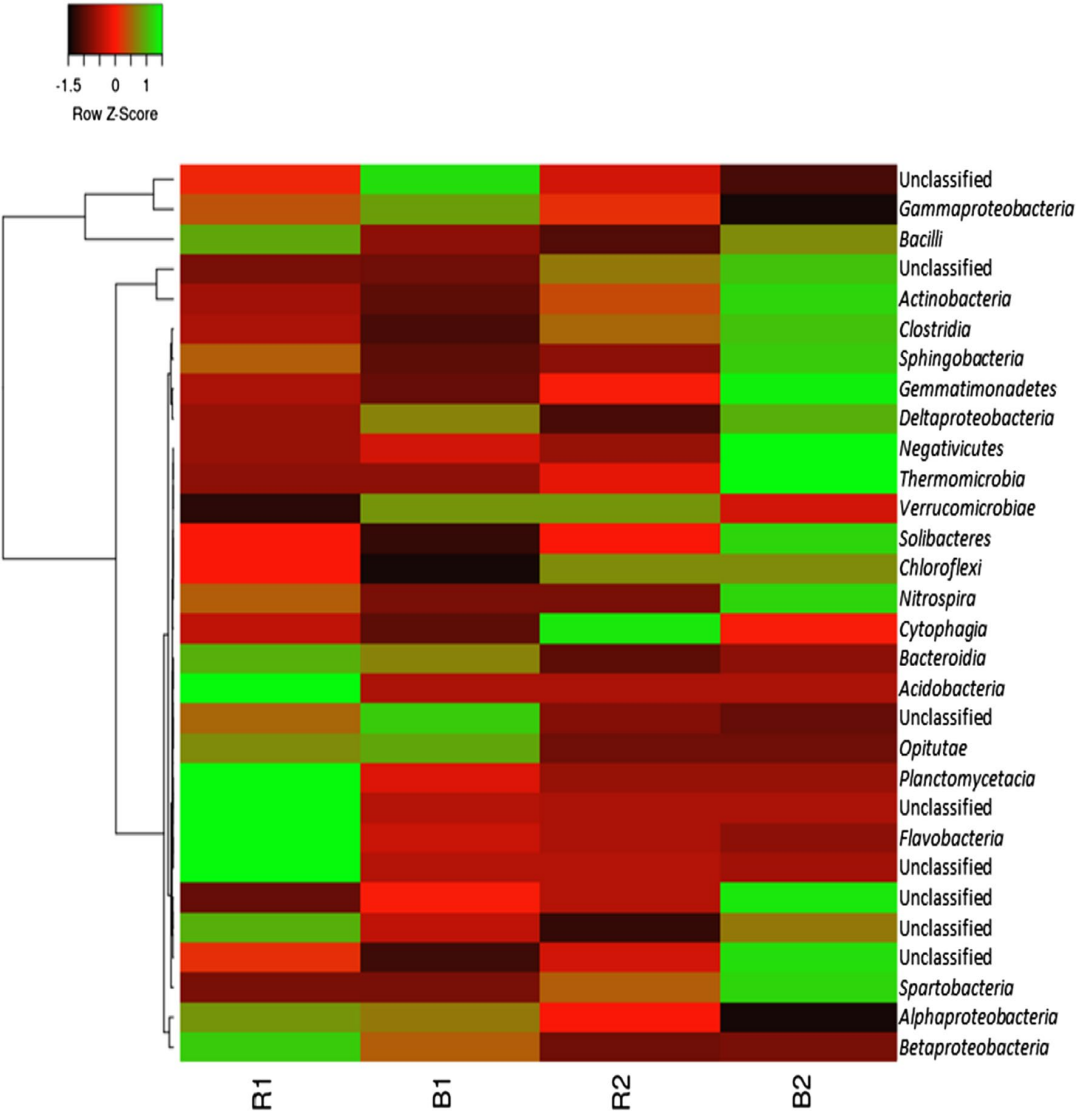
A heat-map of the bacterial taxonomic structure and relative abundance at class level from sunflower rhizosphere and bulk soils. The scale bar signifies colour saturation gradient established by the relative abundances with z-score. Bacterial groups with more intense color have higher relative abundant values. *R1* Ditsobottla rhizosphere soil; *B1* Ditsobottla bulk soil; *R2* Kraaipan rhizosphere soil; *B2* Kraaipan bulk soil

Sequence tags were assigned to rhizosphere and bulk soil samples into diverse taxa via rapid metagenomic annotations using MG-RAST software. At the class level, the relative abundance of bacterial class for each soil sample is presented in Fig. 3. Relative abundance of *Bacilli*, *Bacteroidia*, *Acidobacteria*, *Planctomycetacia*, *Flavobacteria*, and *Betaproteobacteria* were higher in R1. *Gammaproteobacteria* and *Opitutae* were observed to be more abundant in the corresponding bulk soil (B1). Bacilli were higher in all soil samples (R1, B1, R2, and B2) with relative abundance values of 38.18, 30.66, 28.85 and 37.43 respectively. *Gammaproteobacteria* were dominant in R1, B1, and R2 soil samples with relative abundance values of 23.74, 28.36, and 21.96 respectively. While *Actinobacteria* (18.05) were recorded to be more abundant in bulk soil sample (B2). *Cytophagia* was exclusively detected in the rhizosphere soil (R2). Whereas, *Actinobacteria*, *Clostridia*, *Spingobacteria*, *Gemmatimonadetes*, *Deltaproteobacteria*, *Negativicutes*, *Thermamicrobia*, *Solibacteres*, *Nitrospira* and *Spartobacteria* were abundant in the bulk soil sample (B2).

**Fig. 4:**
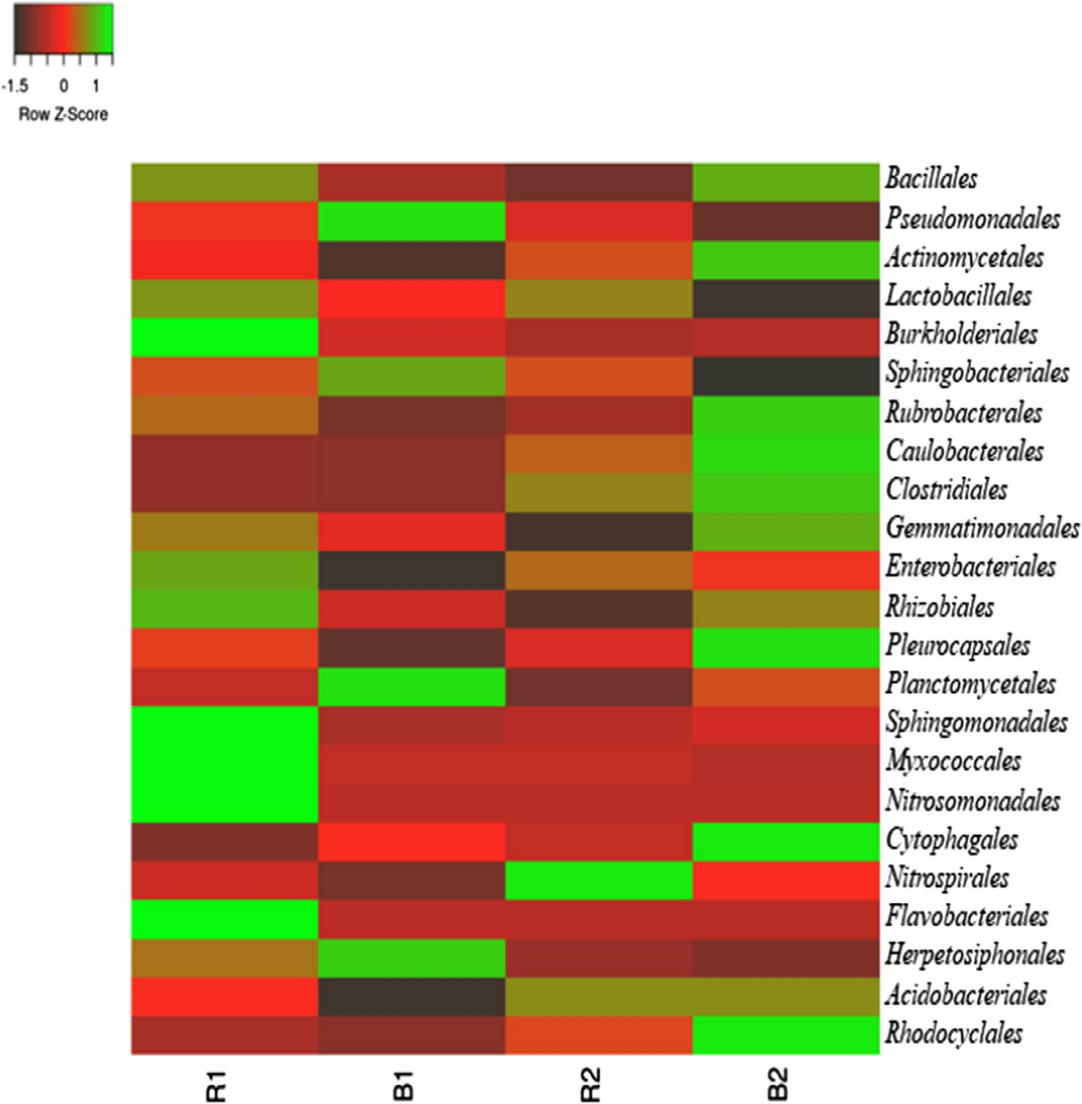
Relative abundance of the bacterial taxonomic structure at order level from sunflower rhizosphere and bulk soils. Bacterial groups with high colour intensity have higher relative abundant values. *R1* Ditsobottla rhizosphere soil; *B1* Ditsobottla bulk soil; *R2* Kraaipan rhizosphere soil; *B2* Kraaipan bulk soil

At the order level, the relative abundance of *Bacillales* were higher in R1 (36.24) than the bulk soil (B1 = 29.28). On the other hand, B2 (37.00) had higher mean relative abundance than the rhizosphere soil (R2 = 26.99). *Pseudomonadales* were higher in bulk soil (B1 = 26.94) than in the R1 (22.64). Also, *Pseudomonadales* in R2 (21.10) were more than those of B2 (7.35). *Actinomycetales* were higher in B2 (7.41) than R1, B1, and R2. From the heatmap Z score color intensity, *Lactobacillales*, *Burkholdeiales*, *Enterobacteriales*, *Rhizobiales*, *Sphingomonadales*, *Myxococcales*, *Nitrosomonadales*, and *Flavobacteriales* were higher in the R1 than in other soil samples. *Spingobacteriale* and *Herpetosiphonales* were relatively more abundant in B2. *Lactobacillales* and *Nitrospirales* were more dominant in R2. *Rubrobacterales*, *Caulobacterales*, *Gemmatimononadales*, *Pleurocapsales*, *Cytophagales* and *Rhodocyclales* were highly abundant in the corresponding bulk soil (B2) (Fig. 4). There were significant differences (p < 0.05) between the mean relative abundance of the bacterial communities of the two farms at the class and order level.

### Effect of soil properties on bacterial communities

**Fig. 5:**
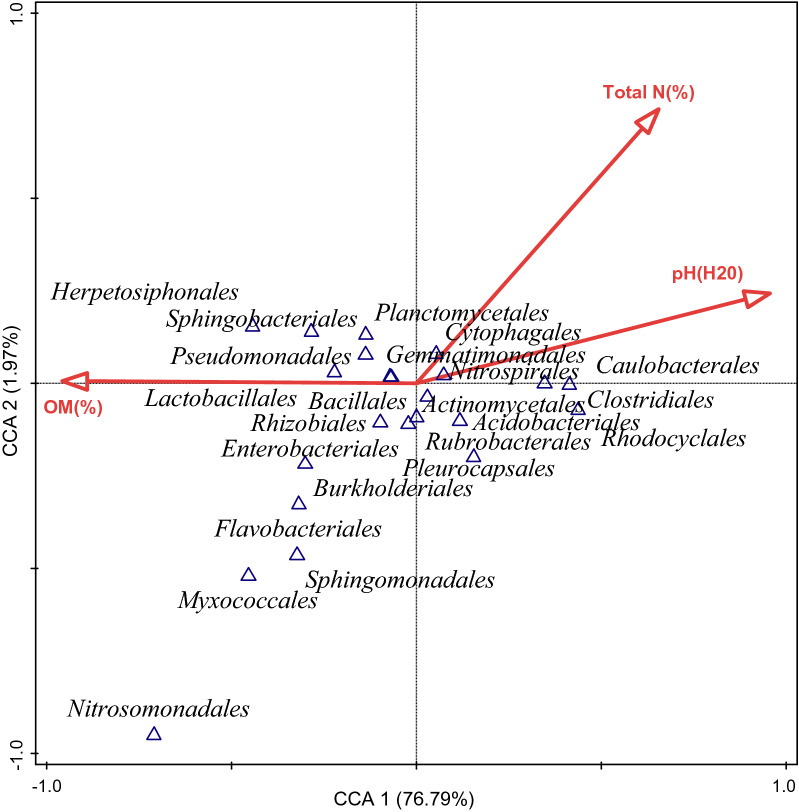
The influence of pH, total N and OM on the bacterial components (at the order level) of sunflower rhizosphere and bulk soils obtained from Ditsobottla and Kraaipan farms using Canonical correspondence analysis. *N* nitrate; *%* percentage; *OM* organic matter; *R1* Ditsobottla rhizosphere soil; *B1* Ditsobottla bulk soil; *R2* Kraaipan rhizosphere soil; *B2* Kraaipan bulk soil

**Fig. 6:**
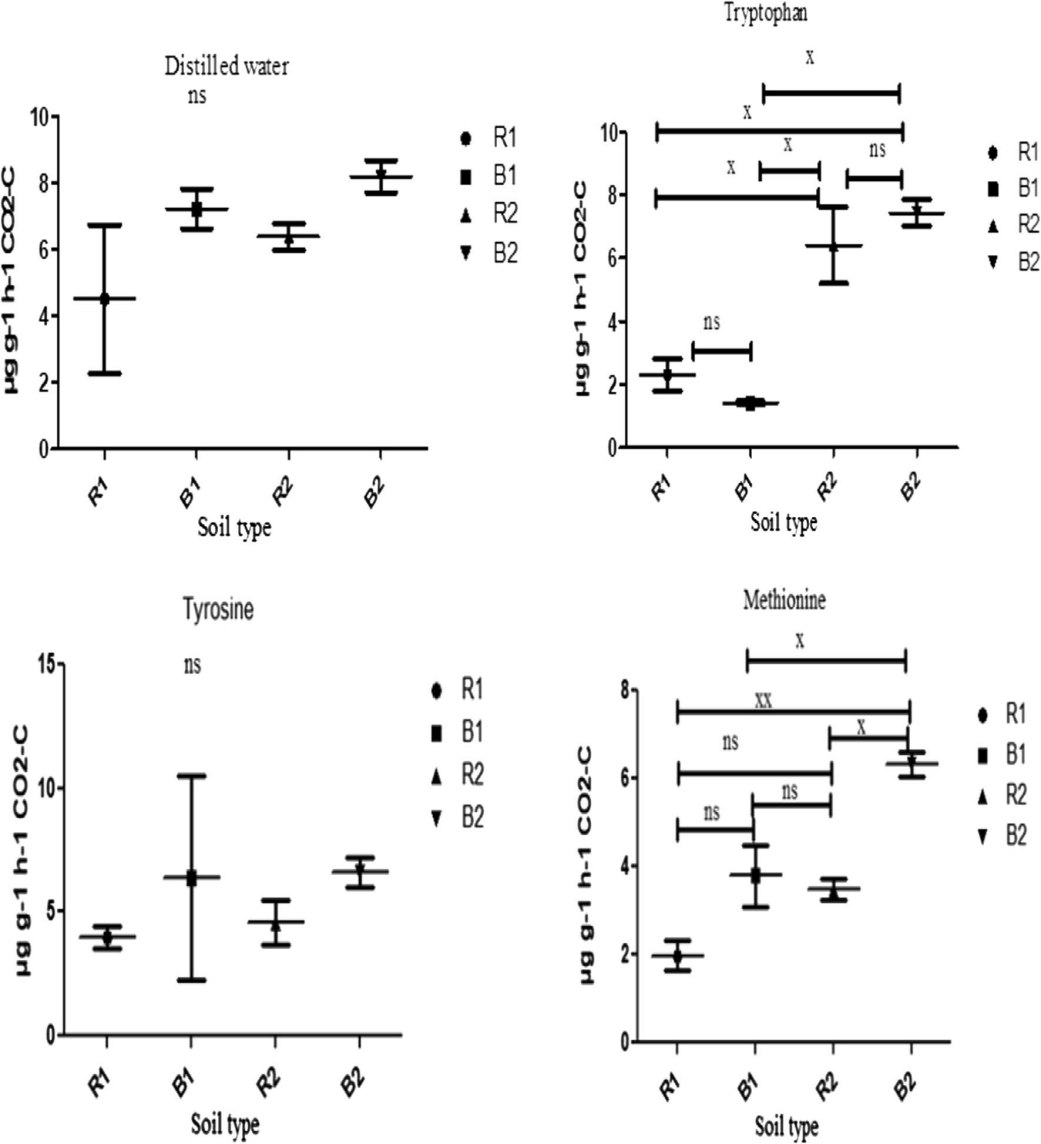
Effects of soil type on soil functional properties as measured for distilled water i.e. control, tryptophan, tyrosine and methionine using MicroResp assay. Number of replicates (n) = 2. Data represent mean ± SE, *ns* not statistically significant; *x* statistically significant; *xx* more statistically significant; *R1* Ditsobottla rhizosphere soil; *B1* Ditsobottla bulk soil; *R2* Kraaipan rhizosphere soil; *B2* Kraaipan bulk soil

The CCA plot showed that the composition of bacterial communities was influenced by the soil properties. The vector length of pH and total N were (on axis 1) positively correlated with *Planctomycetales*, *Cytophagales*, *Gemmatimonadales*, *Nitrospirales* and *Caulobacterales*. On axis 2, the vector length of organic matter positively correlated with *Lactobacillales*, *Bacillales*, *Rhizobiales*, *Enterobacteriales*, *Burkholderiales*, *Flavobacteriales*, *Sphingomonadales*, *Myxococcales*, *Nitrosomonadales* but negatively correlated with Actinomycetales, Clostridiales, *Acidobacteriales*, *Rubrobacterales*, *Pleurocapsales*, and *Rhodocyclales* (Fig. 5). The soil properties that best explained the variance detected in the bacterial community structures observed in Figs. 5 and 6 were ascertained using the forward selection and the Monte Carlo permutation test with random permutations. It was noted that all the soil variables contributed to the difference in the bacterial composition and abundance of the different soil samples, their contributions were, however, not significant (p ≥ 0.05) (Table 1). pH had the highest contribution of 71.4%, followed by total N with 20.0% contribution, and 8.6% contribution from organic matter.

### Carbon substrate utilization by the sunflower rhizosphere soil isolates

**Fig. 7:**
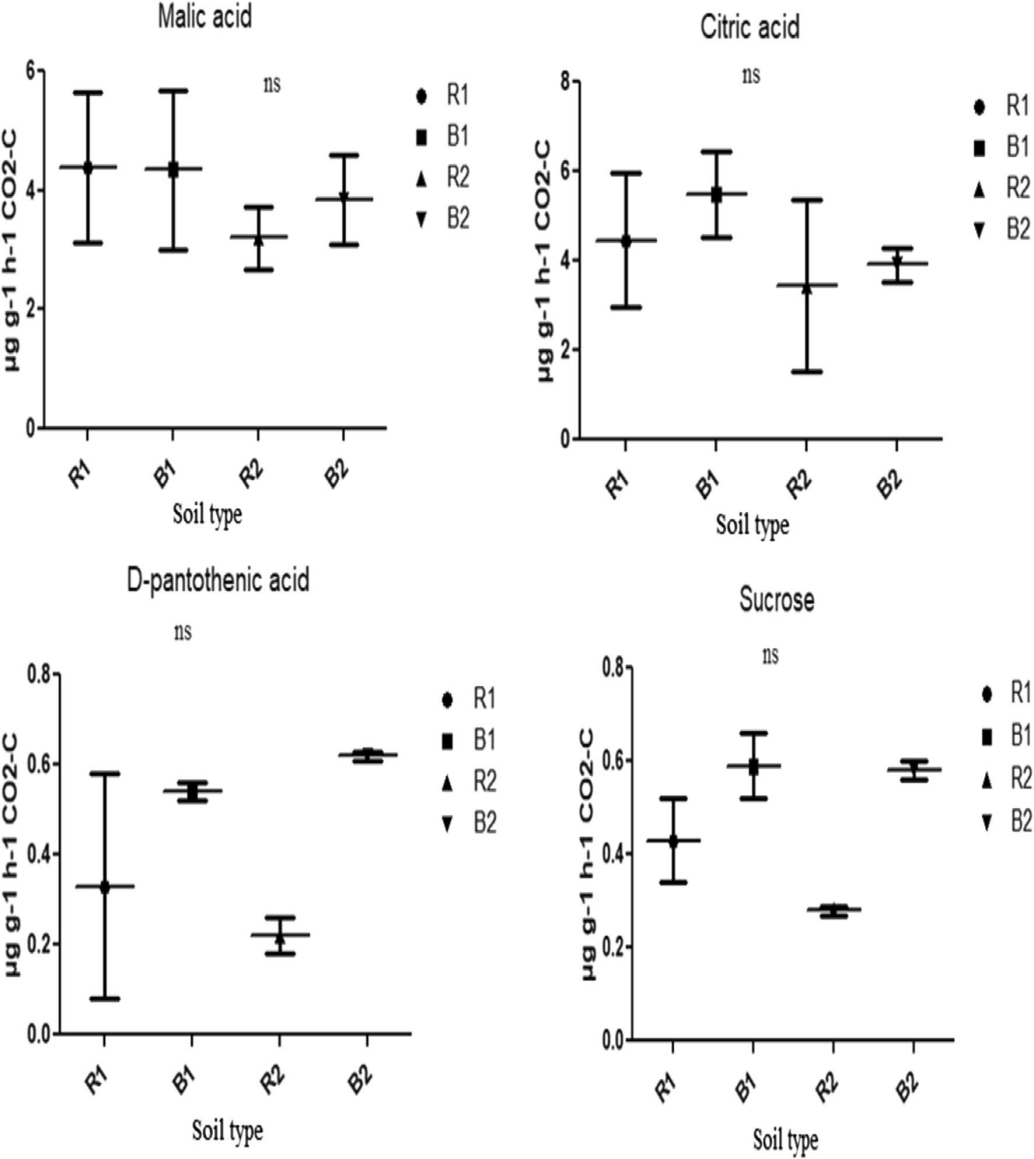
Effects of soil type on soil functional properties as measured for malic acid, citric acid, d-pantothenic acid and sucrose using MicroResp assay. Number of replicates (n) = 2. Number of replicates (n) = 2. Data represent mean ± SE, *ns* not statistically significant; *R1* Ditsobottla rhizosphere soil; *B1* Ditsobottla bulk soil; *R2* Kraaipan rhizosphere soil; *B2* Kraaipan bulk soil

**Fig. 8:**
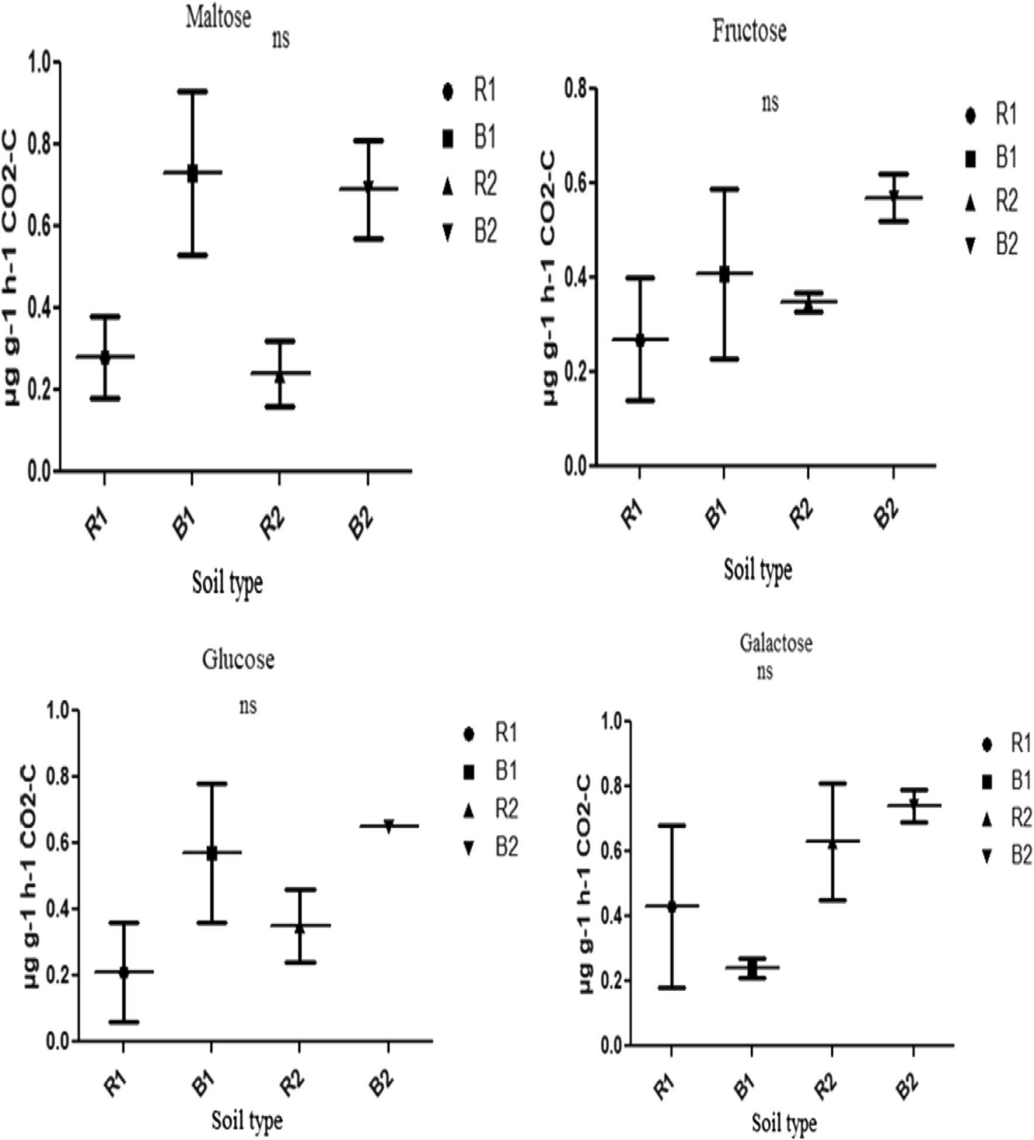
Effects of soil type on soil functional properties as measured for maltose, fructose, glucose and galactose using MicroResp assay. Number of replicates (n) = 2. Data represent mean ± SE, *ns* not statistically significant; *R1* Ditsobottla rhizosphere soil; *B1* Ditsobottla bulk soil; *R2* Kraaipan rhizosphere soil; *B2* Kraaipan bulk soil

Different soil types (R1, B1, R2, and B2) showed different carbon substrate utilization and respiration rates as depicted in Figs. 6, 7 and 8. In tryptophan amended soils, the bacterial community of B2 had the highest respiration rate, which was significantly different (p < 0.05) from those of R1 and B1 (Fig. 6). Similarly, the bacterial community of B2 significantly (p < 0.05) utilize methionine than those of R1, B1 and R2 with a concomitant higher respiration rate as depicted by the amount of CO_2_ produced (Fig. 6). However, the bacterial respiration rates in the soils (that is, R1, B1, R2 and B2) amended with distilled water (control), tyrosine, malic acid, citric acid, d-pantothenic acid, sucrose, maltose, fructose, glucose and galactose showed no significant difference (p > 0.05) (Figs. 6, 7 and 8).

**Fig. 9:**
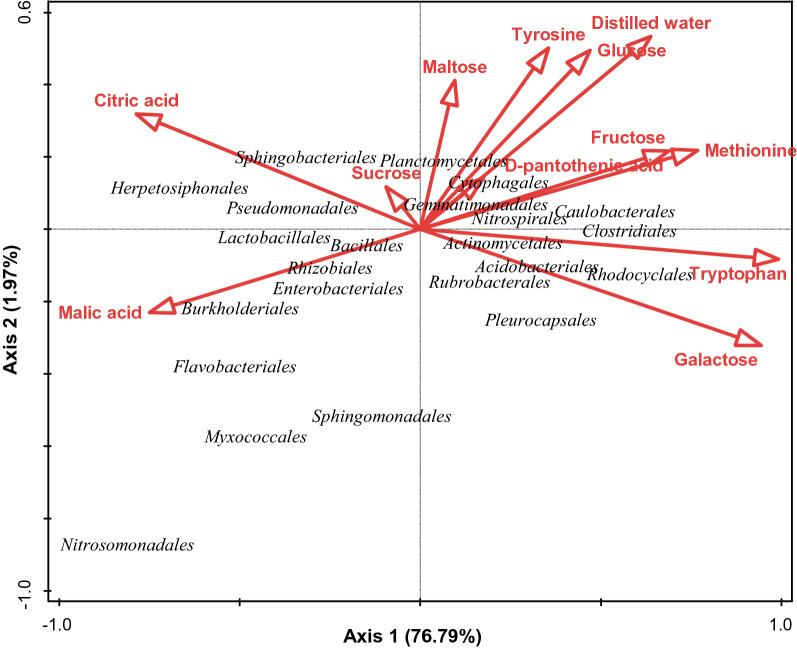
The influence of carbon substrate utilization on the bacterial components (at the order level) of sunflower rhizosphere and bulk soils obtained from Ditsobottla and Kraaipan farms using canonical correspondence analysis

For the two rhizosphere soils, R2 showed a higher total respiration rate (16.83 µg g^−1^ h^−1^ CO_2_–C), while for the two bulk soils, B2 showed a higher total respiration rate (23.80 µg g^−1^ h^−1^ CO_2_–C). The respiration responses from distilled water utilization (basal respiration) were 4.50, 7.23, 6.38 and 8.19 µg g^−1^ h^−1^ CO_2_–C for R1, B1, R2 and B2 respectively.The influence of the carbon substrate utilization on the relative abundance of bacterial species at the order level was determined using CCA (Fig. 9). *Herpetosiphonales*, *Sphingobacteriales*, *Pseudomonadales*, and *Planctomycetales* were positively correlated with citric acid and sucrose, but negatively correlated with tyrosine, glucose, maltose, fructose, galactose tryptophan, methionine, d-pantothenic acid and distilled water. *Cytophagales*, *Gemmatimonadales*, *Nitrospirales*, and *Canulobacterales* were positively correlated with maltose, tyrosine, d-pantothenic acid, methionine, glucose, fructose, and distilled water but negatively correlated with tryptophan, galactose, malic acid, sucrose, and citric acid, while *Actinomycelales*, *Acidobacteriales*, *Rhodocyclales*, *Rubrobacterales*, and *Pleurocapsales* were positively correlated with tryptophan and galactose but negatively correlated with tyrosine, distilled water, glucose, maltose, fructose, malic acid, citric acid, sucrose, methionine and d-pantothenic acid. However, only malic acid influenced the abundance of *Lactobacillales*, *Bacillales*, *Rhizobales*, *Enterobacteriales*, *Burkhoderiales*, *Flavobacteriales*, *Myococcales*, *Nitrosomonadales*, and *Sphingomonadales* (Fig. 9). Furthermore, the effect of sucrose, maltose and d-pantothenic acid on the bacterial community were lower than those of glucose, fructose, malic acid, citric acid, tryptophan, tyrosine, methionine, galactose and distilled water as shown by the lengths of the vectors. The total variation is 0.12110 and permutation test results on all axes presented as pseudo-F < 0.1 and p = 1.

Out of all the carbon substrates used for this study (Fig. 9), tryptophan had the highest contribution of 75.9% (p = 0.102), followed by galactose (16.7%). Thus, from Additional file 2: Table S3, tryptophan best explained (75.9%) the variation that occurred in the soil bacterial (specifically *Actinomycetales*, *Acidobacteriales*, *Rubrobacteriales*, *Rhodocyclales*, *Pleurocapsules*) composition and abundance (Fig. 9). Citric acid, however, had 7.5% effect (p = 1.000) on the soil bacterial components.

From Additional file 1: Fig. S3, the OM positively correlated with malic acid, citric acid, tyrosine and sucrose, but negatively correlated with other carbon substrates. Total N and pH positively correlated with galactose and tryptophan, but negatively correlated with other carbon substrates.

It was noted that all the soil variables contributed to the difference in carbon substrate utilization by the bacterial components of the soils (Additional file 2: Table S4). Organic matter showed 51.4% contribution (p = 0.052) to malic acid, citric acid, tyrosine and sucrose utilization by the bacteria. pH and total N on the other hand, respectively showed 32.5% (p = 0.212) and 16.2% (p = 1.000) contribution (Additional file 2: Table S4) to both galactose and tryptophan utilization by the bacterial components of the soil (Additional file 1: Fig. S3).

## Discussion

In this study, we investigated the influence of soil properties and carbon substrate utilization on the bacterial components and diversity associated with sunflower rhizosphere and bulk soils obtained from two farms with different history of agricultural practices in North West Province of South Africa. MG-RAST analyses of 16S rRNA amplicon sequenced data was done prior to determination of mean relative abundance, alpha/beta diversity. Soil properties were determined as previously described, while the ability of the bacterial communities of the sampled soils to utilize carbon substrates was ascertained by using CLPP.

With respect to the structural bacterial diversity, .the sharp reduction in mean sequence reads of R1, R2, B1 and B2 after QC is due to the exclusion of low quality and contaminated sequences that could have affected downstream analyses (Zhou et al. 2014). Furthermore, analysis using a Venn diagram showed that at the OTU level, the read sequences of R1 and B1 had 34.0% similarity, R2 and B2 had 35.6% similarity, and R1 and R2 had 32.7% similarity (Fig. 1). This result contradicts the result of Molefe et al. (2021), who reported 10.9% bacterial similarity between maize rhizosphere and bulk soils obtained from Ventersdorp, and 17.2% bacterial similarity between maize rhizosphere and bulk soils obtained from Mafikeng in South Africa. The contradiction between the results of the two studies could be due to the different sampling locations and plant type.

The bacterial diversity within each sample, that is, R1, R2, B1 or B2 was determined using different diversity indices such as Equitability_J, Fisher_alpha, Berger-Parker and Chao-1 (Table 1). At a probability level of 0.05, it was observed that the bacterial diversity ‘within’ each sampling site showed significant difference (p = 0.01, R = 0.06).The uniqueness ‘between’ samples (beta diversity) was further determined in this study by PCoA analysis (Fig. 2). At a probability level of 0.05, the analysis revealed significant differences (*p* = 0.01, R = 1.167) in the structural diversity ‘between’ samples. The significant variation in the bacterial diversity observed between samples in this study can partly be attributed to the differences in the agricultural practices (Additional file 2: Table S1) of the two farms. For instance, herbicides, pesticides and bio-fertilizer were applied on Ditsobottla farm but not on Kraaipan farm, and the effects of these components on the bacterial communities of sunflower and other plants rhizospheres have been reported previously (Kostyuchenko et al. 2021; Thokchom et al. 2017; Igiehon et al. 2021; Li et al. 2022). However, chemical fertilizer (NPK 15:8:4), which was applied on both farms, has also been reported to influence changes on the relative abundances of bacterial components of agricultural crop rhizospheres (Li et al. 2020; Ai et al. 2012; Sun et al. 2018). Thus, the cumulative effects resulting from herbicide, pesticide, bio-fertilizer and chemical fertilizer applications on Ditsobottla farm may have triggered the significant bacterial composition variations observed between Ditsobottla farm and Kraaipan farm (which was only applied with only chemical fertilizer).The results of the bacterial structural diversity of sunflower rhizosphere and bulk soils in the two farms revealed different bacterial taxa both at the class and order level. The relative high abundance of the bacteria in R1 may be linked to the exudates from the sunflower roots. Root exudates have been reported to attract bacteria and other biotic components to the rhizosphere, rhizoplane and endosphere of plant roots (Philippot et al. 2013; Gkarmiri et al. 2017; Jacoby et al. 2017). Similarly, the abundance of the bacteria might be traceable to the bio-fertilizer that was previously applied to the soil (Additional file 2: Table S1), since bio-inoculant applications can influence the bacterial diversity of plant rhizosphere (Igiehon et al. 2021; Mayer et al. 2021; Mohammed et al. 2020). Considering the unclassified bacteria, one possibility, is that, the unclassified bacterial group might contain novel bacterial species that have not been previously identified probably because the nutritional compositions for the cultivation of such bacteria are unknown (Enagbonma et al. 2020). The unclassified bacterial group may harbor species with novel plant growth promoting traits that may be useful to improve the growth of sunflower crops, and perhaps, other agricultural crops.

At the order level, among all the bacterial taxa captured in the two farms, *Bacillales* were the most dominant. *Bacillales* was more dominant in R1 and B2 with mean relative abundance values of 36.24 and 37.00 respectively (Fig. 4). *Bacillales* dominance in these soils may be attributed to the fact that members of this group possess heat-resistant components (that is, endospores) in their cellular structures (Filippidou et al. 2016). Possession of endospores is a survival strategy for bacteria to strive in environments with high temperatures (Filippidou et al. 2016; Gray et al. 2019). The two sunflower farms used in this study were located in semi-arid region of South Africa, which is characterized with hot weather conditions because of its relatively low amount of rainfall. Low detection of endospore forming bacteria was, however, recorded in metagenomics datasets using *spo0A* and *gpr* molecular markers because these markers were absent in common environments (such as soil) of these endospore-forming bacteria, with *spo0A* present in the microbiome of mammals (Filippidou et al. 2015).

Furthermore, the positive influence of total N (%) and pH on five bacteria groups at the order level indicates that an increase in the soil total N and pH would lead to a direct increase in the bacterial species abundance of the five taxa. These bacteria were captured from R2 (Fig. 6). From Fig. 5, the abundance of *Lactobacillales*, *Bacillales*, *Rhizobiales*, Enterobacteriales, *Burkholderiales*, *Flavobacteriales*, *Sphingomonadales*, *Myxococcales* and *Nitrosomonadales* obtained from R1 (Additional file 1: Fig. S1) was positively influenced by OM. Organic matter was positively related with these bacterial orders and not others since the components of the organic matter supported the growth and activities of the bacterial orders (Esmaeilzadeh and Ahangar 2014; Mohammadi et al. 2011). In addition, Additional file 1: Fig. S1 revealed that the bacterial species from B1 and B2 were slightly influenced by soil total N, pH and OM when compared to R1 and R2. Therefore, the greater positive influence of soil N, pH and OM on certain bacterial communities of R1 and R2 (rhizosphere soils of Ditsobottla and Kraaipan farms respectively) may be connected to the high biological activities that might have occurred in the root regions of the sunflower plants (Philippot et al. 2013). For instance, increase in OM resulting from the decomposition of debris (Kotroczó et al. 2014; Shahbaz et al. 2017) might have led to the proliferation of *Planctomycetales*, *Cytophagales*, *Gemmatimonadales*, *Nitrospirales* and *Caulobacteriales* observed in R1. Although, the physiochemical parameter that mostly contributed and best explained the differences in bacterial communities was soil pH (Additional file 2: Table S2) and the pH showed 71.4% (p = 0.184) contribution to the bacterial community profile of *Planctomycetales*, *Cytophagales*, *Gemmatimonadales*, *Nitrospirales* and *Caulobacteriales.*

In addition, the community respiration among various groups of microorganisms has made it possible to measure their functions in the ecosystem (Di Salvo et al. 2018). The functionality of bacterial community of soil samples from the two farms was further ascertained by determining their ability to utilize different carbon substrates using CLPP technique (Figs. 6, 7 and 8). Increase in carbon substrate utilization is directly proportional to increase in CO_2_ released or produced (respiration rate) (Creamer et al. 2016). Carbon substrates have huge ability to distinguish diverse soil bacterial communities through carbon mineralization capacity (Amoo et al. 2021). Bacterial communities of R1, B1, R2 and B2 soils were found to use the different carbon substrates as an energy source, but significant differences were only observed in tryptophan and methionine amended soils. In particular, in tryptophan amended soils, the bacterial components of R2 and B2 showed significant (p < 0.05) higher carbon substrate utilization and respiration rates than those of R1 and B1 (Fig. 6). Also, in soils amended with methionine, bacterial components of B2 significantly (p < 0.05) utilized the carbon substrate and produced more CO_2_ than those of R1, B1 and R2 (Fig. 6).

The total respiration rate resulting from the summation of the 11 carbon substrates without basal respiration (Additional file 1: Fig. S2) was further extrapolated. The quantity of carbon substrate utilized shows the abundance of the microbial communities that are able to utilize a particular carbon source (Creamer et al. 2016). The higher the total quantity of carbon source utilized, the greater the metabolic properties and functional diversity of the bacterial communities (Jones et al. 2018; Deng et al. 2011). From the extrapolation, our results showed that rhizosphere soils had higher respiration rate compared with the basal respiration. However, the higher microbial activity (respiration function) observed in the bulk soil (B2) may have been caused by the contributions of extraneous abiotic factors in B2, since it was reported by Leckie et al. (2004); Hannam et al. (2006); He et al. (2006) that the activities (e.g. respiration) of soil microbial communities are not only affected by biotic factors but also by the abiotic components of the soil.

In addition, it was observed that utilization of most of the carbon substrates positively influenced bacterial abundance and composition. In particular, the organic acid (citric acid) and a disaccharide (sucrose) had a positive influence on *Herpetosiphonales*, *Sphingobacteriales*, *Pseudomonadales*, and *Planctomycetales* (Fig. 9). Citric acid is an intermediate of the Krebs cycle, which is a chain of biochemical reactions utilized by aerobic organisms to produce energy in the form of ATP via the oxidation of acetate (obtained from carbohydrate, fatty acid and protein oxidation) (Gasmi et al. 2021; Kumar and Dubey 2019).

The carbon substrate that best explained these differences in bacterial components was an essential amino acid (tryptophan) (Additional file 2:  Table S3). This amino acid showed 75.9% (p = 0.102) contribution to the bacterial profile of *Actinomycelales*, *Acidobacteriales*, *Rhodocyclales*, *Rubrobacterales*, and *Pleurocapsales* (Fig. 9). Also, the influence of OM, total N and pH on carbon substrate utilization was investigated. It was observed that OM of the sunflower soils positively had influence on the utilization of malic acid, citric acid, tyrosine and sucrose utilization by the soil bacterial species, while the utilization of disaccharide (galactose) and essential amino acid (tryptophan) by the soil bacterial species was positively influenced by both the soil total N and pH (Additional file 1: Fig. S3). The soil parameter that best explained the differences in soil bacterial utilization of carbon substrates was OM. Specifically, soil OM showed 51.4% contribution (p = 0.052) to the carbon substrate utilization ( Additional file 2: Table S4). However, soil OM, total N and pH did not positively influence bacterial utilization of other carbon substrates such as methionine, fructose, glucose, maltose, d-pantothenic acid and distilled water (Additional file 1: Fig. S3).

Finally, at a probability level of 0.05, significant differences were observed for the alpha and beta diversity of the soil bacterial communities. At the class level, *Bacilli*, *Bacteroidia*, *Acidobacteria*, *Planctomycetacia*, *Flavobacteria*, and *Betaproteobacteria* were relatively higher in R1 than the corresponding bulk soil samples (B1), while at the order level, among all the bacterial taxa captured in the two farms, *Bacillales* were the most dominant. Bacterial communities of R1, B1, R2 and B2 soils were found to use the different carbon substrates as an energy source, but significant differences were only observed in tryptophan and methionine amended soils. The utilization of most of the carbon substrates positively influenced the soil bacterial communities. The presence of unclassified bacteria in this study, calls for a further effort to capture and identify these unknown bacteria, since it may be possible to discover novel bacterial species with plant growth promoting functionality.

## Supplementary Information

**Additional file 1: Figure S1.** The influence of pH, total N (%) and OM (%) on sunflower rhizosphere and bulk soils obtained from Ditsobottla and Kraaipan farms using Canonical correspondence analysis. *N* nitrate; *%* percentage; *OM* organic matter; *R1* Ditsobottla rhizosphere soil; *B1* Ditsobottla bulk soil; *R2* Kraaipan rhizosphere soil; *B2* Kraaipan bulk soil. **Figure S2.** Effects of soil type on soil functional properties as measured for distilled water (basal respiration) and sum of the individual respiration rate of the 11 carbon substrates (total respiration) used in the CLPP assay. Number of replicates (n) = 2. *R1* Ditsobottla rhizosphere soil; *B1* Ditsobottla bulk soil; *R2* Kraaipan rhizosphere soil; *B2* Kraaipan bulk soil. **Figure S3.** The influence of pH, total N (%) and OM (%) on carbon substrate utilization by bacterial components of sunflower rhizosphere and bulk soils obtained from Ditsobottla and Kraaipan farms using canonical correspondence analysis. *N* nitrate; *%* percentage; *OM* organic matter.

**Additional file 2: Table S1.** Agricultural history of Ditsobottla and Kraaipan farms. **Table S2.** Forward selection of soil physio-chemical components that best described difference in bacterial components between sunflower rhizosphere and bulk soils of Ditsobottla and Kraaipan farms. **Table S3.** Forward selection of soil carbon substrates that best described difference in bacterial components between sunflower rhizosphere and bulk soils of Ditsobottla and Kraaipan farms. **Table S4.** Forward selection of soil physio-chemical components that best described difference in carbon substrate utilization between sunflower rhizosphere and bulk soils of Ditsobottla and Kraaipan farms.

## Data Availability

The datasets are available in NCBI database under Bioproject ID PRJNA672856.
