# Supplementary material for: Effects of soil properties and carbon substrates on bacterial diversity of two sunflower farms
Source: AMB Express. 2022 Apr 23;12:47. doi: 10.1186/s13568-022-01388-9 (PMC9035202; doi:10.1186/s13568-022-01388-9)
Supplement: Supplementary file 1 — Additional file 1: Figure S1. The influence of pH, total N (%) and OM (%) on sunflower rhizosphere and bulk soils obtained from Ditsobottla and Kraaipan farms using Canonical correspondence analysis. N nitrate; % percentage; OM organic matter; R1 Ditsobottla rhizosphere soil; B1 Ditsobottla bulk soil; R2 Kraaipan rhizosphere soil; B2 Kraaipan bulk soil. Figure S2. Effects of soil type on soil functional properties as measured for distilled water (basal respiration) and sum of the individual respiration rate of the 11 carbon substrates (total respiration) used in the CLPP assay. Number of replicates (n) = 2. R1 Ditsobottla rhizosphere soil; B1 Ditsobottla bulk soil; R2 Kraaipan rhizosphere soil; B2 Kraaipan bulk soil. Figure S3. The influence of pH, total N (%) and OM (%) on carbon substrate utilization by bacterial components of sunflower rhizosphere and bulk soils obtained from Ditsobottla and Kraaipan farms using canonical correspondence analysis. N nitrate; % percentage; OM organic matter. [file 13568_2022_1388_MOESM1_ESM.docx]

**Supplementary figures**

** Fig. S1:** The influence of pH, total N (%) and OM (%) on sunflower rhizosphere and bulk soils obtained from Ditsobottla and Kraaipan farms using Canonical correspondence analysis. N – nitrate, % - percentage, OM – organic matter, R1- Ditsobottla rhizosphere soil, B1- Ditsobottla bulk soil, R2- Kraaipan rhizosphere soil, B2- Kraaipan bulk soil

**Fig. S2:** Effects of soil type on soil functional properties as measured for distilled water (basal respiration) and sum of the individual respiration rate of the 11 carbon substrates (total respiration) used in the CLPP assay. Number of replicates (n) = 2. R1= Ditsobottla rhizosphere soil, B1= Ditsobottla bulk soil, R2= Kraaipan rhizosphere soil, B2 = Kraaipan bulk soil.

 **Fig. S3**: The influence of pH, total N (%) and OM (%) on carbon substrate utilization by bacterial components of sunflower rhizosphere and bulk soils obtained from Ditsobottla and Kraaipan farms using canonical correspondence analysis. N – nitrate, % - percentage, OM – organic matter
