# Supplementary material for: Effects of soil properties and carbon substrates on bacterial diversity of two sunflower farms
Source: AMB Express. 2022 Apr 23;12:47. doi: 10.1186/s13568-022-01388-9 (PMC9035202; doi:10.1186/s13568-022-01388-9)
Supplement: Supplementary file 2 — Additional file 2: Table S1. Agricultural history of Ditsobottla and Kraaipan farms. Table S2. Forward selection of soil physio-chemical components that best described difference in bacterial components between sunflower rhizosphere and bulk soils of Ditsobottla and Kraaipan farms. Table S3. Forward selection of soil carbon substrates that best described difference in bacterial components between sunflower rhizosphere and bulk soils of Ditsobottla and Kraaipan farms. Table S4. Forward selection of soil physio-chemical components that best described difference in carbon substrate utilization between sunflower rhizosphere and bulk soils of Ditsobottla and Kraaipan farms. [file 13568_2022_1388_MOESM2_ESM.docx]

Table S1. Agricultural history of Ditsobottla and Kraaipan farms

| Agricultural practices | Farm | |
| --- | --- | --- |
|  | Ditsobottla sunflower farm | Kraaipan sunflower farm |
| Herbicides application before planting | Yes (Roundup: Glyphosate-based herbicide) | No |
| Herbicides application after planting | Yes (Metagan Gold) | No |
| Bio-fertilizer | Yes (Omina fertilizer) | No |
| Pesticides | Yes (Judo 50EC) | No |
| Fertilizer | Yes (NPK15:8:4) | Yes (NPK15:8:4) |
| Organic manure | No | No |
| Mechanized | Yes( Ploughing and Tillage) | Yes (Mowing, Ploughing, Tillage) |

Table S2. Forward selection of soil physio-chemical components that best described difference in bacterial components between sunflower rhizosphere and bulk soils of Ditsobottla and Kraaipan farms

| Environmental variable | Explains % | Contribution % | Pseudo-F | *P* |
| --- | --- | --- | --- | --- |
| pH | 71.4 | 71.4 | 5.0 | 0.184 |
| Total N (%) | 20.0 | 20.0 | 2.3 | 0.218 |
| OM (%) | 8.6 | 8.6 | <0.1 | 1. |

Legend: N – nitrate, % - percentage, OM – organic matter, p – probability value

Table S3: Forward selection of soil carbon substrates that best described difference in bacterial components between sunflower rhizosphere and bulk soils of Ditsobottla and Kraaipan farms

| **Carbon substrate** | **Explained %** | **Contribution %** | **Pseudo-F** | **P** |
| --- | --- | --- | --- | --- |
| Tryptophan | 75.9 | 75.9 | 6.3 | 0.102 |
| Galactose | 16.7 | 16.7 | 2.2 | 0.286 |
| Citric acid | 7.5 | 7.5 | <0.1 | 1. |

Legend: % - percentage, p – probability value

Table S4: Forward selection of soil physio-chemical components that best described difference in carbon substrate utilization between sunflower rhizosphere and bulk soils of Ditsobottla and Kraaipan farms

| PC | Explain % | Contribution % | Pseudo-F | P- |
| --- | --- | --- | --- | --- |
| OM (%) | 51.4 | 51.4 | 2.1 | 0.052 |
| pH (N/A) | 32.5 | 32.5 | 2.0 | 0.212 |
| Total N (%) | 16.2 | 16.2 | <0.1 | 1.000 |

Legend: PC – physiochemical components, N – nitrate, % - percentage, OM – organic matter, p – probability value
